# A Deep Learning-Based Emergency Alert Wake-Up Signal Detection Method for the UHD Broadcasting System

**DOI:** 10.3390/s24134162

**Published:** 2024-06-26

**Authors:** Jin-Hyuk Song, Myung-Sun Baek, Byungjun Bae, Hyoung-Kyu Song

**Affiliations:** 1 Department of Information and Communication Engineering, Sejong University, Seoul 05006, Republic of Korea; song2478155@gmail.com; 2 Electronics and Telecommunications Research Institute (ETRI), Daejeon 34129, Republic of Korea; sabman@etri.re.kr (M.-S.B.); 1080i@etri.re.kr (B.B.); 3 Department of Convergence Engineering for Intelligent Drone, Sejong University, Seoul 05006, Republic of Korea

**Keywords:** deep learning, emergency alert, wake-up signal detection, ATSC 3.0, UHD broadcasting

## Abstract

With the increasing frequency and severity of disasters and accidents, there is a growing need for efficient emergency alert systems. The ultra-high definition (UHD) broadcasting service based on Advanced Television Systems Committee (ATSC) 3.0, a leading terrestrial digital broadcasting system, offers such capabilities, including a wake-up function for minimizing damage through early alerts. In case of a disaster situation, the emergency alert wake-up signal is transmitted, allowing UHD TVs to be activated, enabling individuals to receive emergency alerts and access emergency broadcasting content. However, conventional methods for detecting the bootstrap signal, essential for this function, typically require an ATSC 3.0 demodulator. In this paper, we propose a novel deep learning-based method capable of detecting an emergency wake-up signal without the need for an ATSC 3.0. The proposed method leverages deep learning techniques, specifically a deep neural network (DNN) structure for bootstrap detection and a convolutional neural network (CNN) structure for wake-up signal demodulation and to detect the bootstrap and 2 bit emergency alert wake-up signal. Specifically, our method eliminates the need for Fast Fourier Transform (FFT), frequency synchronization, and interleaving processes typically required by a demodulator. By applying a deep learning in the time domain, we simplify the detection process, allowing for the detection of an emergency alert signal without the full suite of demodulator components required for ATSC 3.0. Furthermore, we have verified the performance of the deep learning-based method using ATSC 3.0-based RF signals and a commercial Software-Defined Radio (SDR) platform in a real environment.

## 1. Introduction

Recently, the scale and frequency of disasters and accidents have increased, leading to a significant rise in damages. To enhance efficient disaster preparedness and expedite damage relief, various broadcasting and communication systems develop emergency alert technologies [1,2,3,4,5,6]. The ATSC (Advanced Television Systems Committee) 3.0 standard is defined as the state-of-the art terrestrial digital broadcasting system specification [7,8] and one of the systems that offers disaster services. The ATSC 3.0 standard includes a wake-up function for emergency alerts [7]. This feature enables devices in standby or sleep mode to recognize emergency alert signals and automatically awaken to deliver emergency messages to individuals.

To enhance spectral efficiency and robustness, the ATSC 3.0 standard employs various improved physical layer techniques, including orthogonal frequency division multiplexing (OFDM) with low-density parity-check (LDPC), layered division multiplexing (LDM), and others [8]. Moreover, the ATSC 3.0 broadcasting standard includes the advanced emergency alert (AEA) information service, which is a pivotal element. To provide the AEA information service, the ATSC 3.0 physical layer system transmits the emergency alert wake-up signal through a bootstrap signal. In case of the disaster situation, the emergency alert wake-up signal is transmitted, and the UHD TV can be woken up and activated after receiving the wake-up signal. As a result, people can receive the emergency alert and watch the emergency broadcasting contents through the activated TV. This service creates significant new value for broadcasters and authorities, as well as viewers in disaster situation [9].

### 1.1. Related Works

ATSC 3.0 utilizes a frame structure composed of three primary components: the bootstrap, preamble, and one or more subframes. The bootstrap provides a universal entry point into a broadcast waveform and has been designed to be a very robust signal and receptacle even at low signal levels [7,8,9]. The bootstrap consists of four OFDM symbols. The initial OFDM symbol within the bootstrap signal is identical across all transmission frames and features repetition patterns in the time domain. Using these characteristics, signal discovery, coarse synchronization, and initial channel estimation are performed. The other symbols contain an emergency alert wake up field as well as system configuration parameters, respectively. The wake-up signal composes of two bits, with each bit located in bootstrap symbols 1 and 2, respectively. Accordingly, some intricate decoding algorithms have been proposed to ensure reliable decoding of the wake-up bits at a low signal-to-noise ratio (SNR) levels [10,11]. In recent times, a machine learning method based on neural networks (NN) has garnered significant attention in the field of wireless communication [12,13,14,15,16,17]. In particular, the references [18,19,20] cover reception method based on baseband signal. Reference [18] proposed a deep learning-based signal detection technique for the multiple-input multiple-output (MIMO) system with multipath fading channel, and the proposed technique can achieve a performance very similar to an optimal algorithm-based detection technique. Reference [19] demonstrates the use of two-layer neural networks for pilot design and deep neural networks (DNN) for channel estimation, achieving superior performance compared to traditional estimation techniques. Furthermore, reference [20] designed and implemented a convolutional neural network (CNN)- and DNN-based approach to model the non-linear transfer function between MIMO transmitters and receivers. Simulation results of the proposed technique show better robustness can be achieved in a condition of an imperfect channel knowledge compared with conventional algorithms. Lastly, in [21], the DNN structure is studied for ATSC 3.0 and can detect only the first bootstrap symbol even under low SNR levels.

### 1.2. Motivation and Contribution

The fundamental concern of the paper is to detect the wake-up signal after the synchronization bootstrap symbol. Conventional bootstrap detection methods’ emergency alert systems often rely on a full ATSC 3.0 demodulator. This requirement limits the applicability of these methods, particularly in scenarios where a full demodulator might not be readily available (e.g., low-power devices or disaster situations). Additionally, conventional methods typically involve complex signal processing techniques like Fast Fourier Transform (FFT), frequency synchronization, and interleaving, which can be computationally expensive and decrease overall system efficiency. Therefore, a new deep learning-based wake-up signal detection method that solves the limitations identified above is needed. Although the study conducted in [21] has demonstrated significant potential, there are several aspects that could further improve the performance of bootstrap detection. Compared to [21], the additional research conducted can be summarized as follows:

- Improve DNN structure by adding four fully connected (FC) layer to learn high-dimensional complex patterns and features.
- Reconstruct an appropriate training dataset to achieve high detection performance in wireless channel and prevent overfitting.
- Optimize received complex signal data used as an input DNN structure.

In this paper, a deep learning-based wake-up signal detection method is proposed for terrestrial UHD emergency alert service based on ATSC 3.0. The proposed method comprises two functional blocks. The initial block focuses on bootstrap signal detection and offset estimation. Within this bootstrap detection block, the first bootstrap symbol is identified, and the time-offset value of the received first bootstrap symbol is estimated. The second block is the emergency wake-up signal detection block, capable of detecting and demodulating the wake-up bits using a deep learning method. Both DNN and CNN structures are considered for bootstrap detection and emergency wake-up signal detection. DNN is well-suited for tasks requiring time synchronization due to their ability to handle temporal sequences effectively. The proposed DNN structure comprises multiple layers of neurons, allowing it to learn and extract these temporal features from the received signal. CNNs are effective in spatial data processing and analysis, making them suitable for wake-up bit detection. Wake-up bits are embedded within the bootstrap signal and can be identified by examining spatial patterns within the signal. Therefore, the proposed method can be efficiently used in a serious disaster situation to announce emergency alert contents. In addition, the proposed method can detect an emergency alert signal without an ATSC 3.0 demodulator, such as a UHD TV, making it efficiently applicable to various systems. This paper is organized as follows: Section 2 describes bootstrap generation and the structure of the ATSC 3.0 standard. The proposed deep learning-based bootstrap detection method is described in Section 3. In Section 4, simulation results and discussions are presented. Section 5 concludes this paper.

## 2. Bootstrap Generation and Structure of ATSC 3.0 Standard

**Figure 1:**
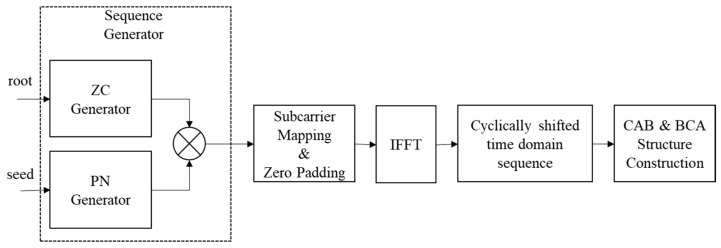
Functional block diagram of bootstrap generator.

The bootstrap facilitates the provision of multiple wireless-based services via time multiplexing within a single RF channel. Serving as a low-level indicator to support these services, it allows a receiver to detect and identify the bootstrap signal. Consequently, this identification provides guidance on accessing the services available through a signal [9]. The bootstrap serves as a universal entry point into an ATSC 3.0-based broadcast waveform, employing a fixed configuration universally recognized by all receiver devices. It carries essential information to facilitate the processing and decoding of the signal associated with a detected bootstrap. In Figure 1, the functional block diagram of the bootstrap generator is presented. Initially, the sequence generator combines a Zadoff–Chu (ZC) sequence with a pseudo-noise (PN) sequence, which includes major and minor versions. The resulting sequence is then transformed into a time-domain sequence through Inverse Fast Fourier Transform (IFFT). After IFFT, cyclic shifts in the time domain are performed to convey information signaled via the bootstrap symbols.

**Figure 2:**
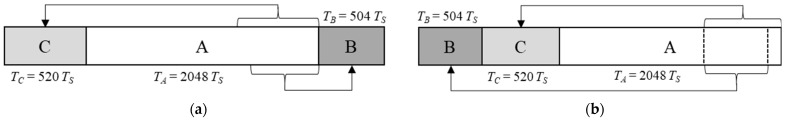
Bootstrap structure in the time domain: (**a**) CAB structure; (**b**) BCA structure.

Finally, the cyclically shifted sequence is transformed into two structures (CAB and BCA) as shown in Figure 2. The initial symbol (bootstrap symbol 0: BS_0) specifically designed for synchronization detection employ the CAB variant. Subsequently, the remaining bootstrap symbols conform to the BCA variant. In the CAB structure, part B consists of the last 504 samples of part A with a frequency and phase shift applied to the originating frequency domain sequence, while part C consists of the last 520 samples of part A. In the BCA structure, part C consists of the last 520 samples of part A, but part B consists of the first 504 samples of part C with a frequency shift applied to the originating frequency domain sequence. 

The complete bootstrap comprises 24 signaling bits. With the exception of the initial bootstrap symbol, each symbol contains essential signaling information. This information encompasses parameters crucial for the configuration of the ATSC 3.0 system, as well as OFDM parameters essential for preamble demodulation. Table 1 shows the signaling information incorporated in the bootstrap, along with the corresponding allocation of bits for each piece of information.

The concatenation of these two bits results in the formation of a 2 bit value representing emergency wake-up information. Consequently, four distinct states emerge, and the presence of at least one ‘1’ in this concatenated value signifies the reception of an emergency alert message. The precise meaning of the wake-up field, as defined in [7], is as follows.

- 00: No active emergency message.
- 01, 10, and 11: Rotating through these values will inform the receiver that there is either a new emergency message or that there is new and substantial information being added to an existing message.

**Figure 3:**
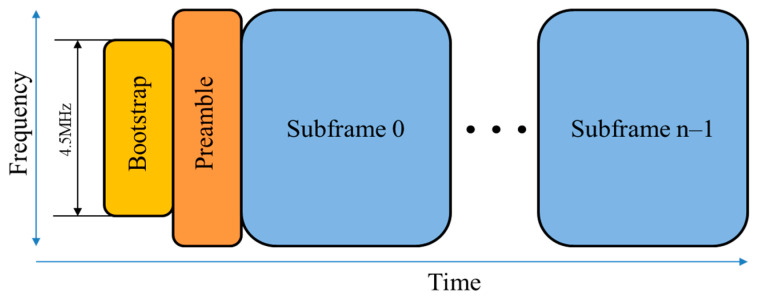
ATSC 3.0 frame structure.

The channel bandwidth of payloads within ATSC 3.0 systems typically occupies 6 MHz. However, the bootstrap symbols exhibit a fixed bandwidth of 4.5 MHz, centered within the RF channel. The system operates at a sampling frequency of 6.144 Msamples/s, employing an FFT size of 2048. This configuration results in a subcarrier spacing of 3 kHz, as illustrated in Figure 3 and Table 2. 

In the UHD broadcasting signal transmission, bootstrap symbols are firstly transmitted. In the time domain, the received bootstrap signal can be written as follows:

(1) $$y_{n}=h\times s_{n}+w_{n}$$

where **h** is the channel impulse response which captures the distortion and delay introduced by the transmission channel. The delay is determined by the number of taps in the filter with length (L), and finally **h** is defined as $\left[h\left(0\right)\;h\left(1\right)\cdots \;h(L-1)\right]$. $s_{n}=[s_{n}\left(0\right)\;s_{n}\left(1\right)\cdots \;s_{n}(3071)]$ is *n*-th bootstrap symbol where $s_{n}\left(k\right)$ is *k*-th sample of *n*-th bootstrap symbol; finally, $w_{n}$ represents additive white Gaussian noise (AWGN) and has the same dimensions as $y_{n}$ and $s_{n}$. It has the constant power spectral density (PSD) across all frequencies and the noise samples are independent of $y_{n}$ and $s_{n}$.

## 3. Proposed Method

The process of the proposed deep learning-based emergency alert wake-up signal detection method is described in Algorithm 1, and the detailed method is described in subsection.
**Algorithm 1** Emergency alert wake-up signal detection method**Input**: The received 3072 complex samples **Output**: Wake-up 2 bits (00, 01, 10, and 11)1: **if** bootstrap detection is False: 2:  go back to step 1 using next received 3072 complex samples 3: **else:**
4:  symbol time offset estimation and compensation 5.  acquisition of time-synchronized 2nd and 3rd bootstrap symbols 6:  demodulation emergency wake-up bits 7:  **if** any wake-up bit is 1: 8:   occur emergency disaster situations 9:   wake-up any connected device 10:   go back to step 6 and demodulation the next bootstrap symbols 11:  **else:** 12:   go back to step 6 and demodulation the next bootstrap symbols 13:  **end if** 14: **end if**

### 3.1. Bootstrap Detection Method Based on Deep Learning Structure

**Figure 4:**
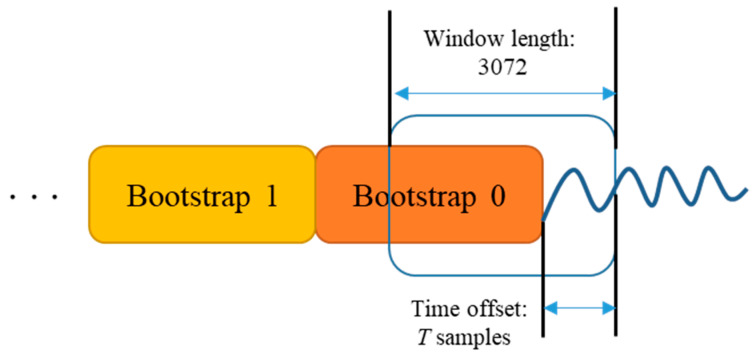
Extraction of the 1st bootstrap symbol with *T* sample time-offset.

For the bootstrap detection based on deep learning technology, 3072 samples are extracted and inserted to the bootstrap detector. These received samples are the baseband signal obtained by receiving RF and down-converting it. During the extraction process, the received first bootstrap symbol with time offset can be written as follows:

(2) $$y_{0}=h*s_{0}^{\prime }+w_{0}$$

where $s_{0}^{\prime }$ is the 1st bootstrap symbol with time offset as follows:

(3) $$s_{0}^{\prime }=[d\left(1\right)\;d(2)\cdots \;d\left(T\right)\;s_{0}\left(0\right)\cdots s_{0}(3071-T)]$$

where *T* is time offset value and *d* denotes payloads signal of the previous subframe. Figure 4 illustrates the extraction process of the 1st bootstrap symbol $s_{0}^{\prime }$ with T time offset. As depicted in Figure 4 and expressed in (3), during the bootstrap detection, $s_{0}^{\prime }$ is extracted with a length of 3072 samples. The extracted bootstrap symbol is subsequently inserted into the proposed deep learning-based bootstrap detector.

**Figure 5:**
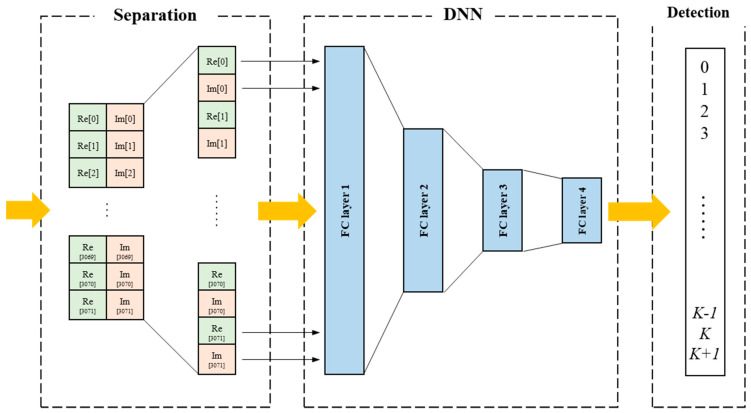
Proposed bootstrap symbol detector based on DNN.

Figure 5 describes the proposed deep learning-based bootstrap detector, comprising a signal separation, a DNN, and a detection part. The extracted bootstrap symbol is inherently in complex signal format and not suitable for direct integration into the DNN-based deep learning system. Therefore, in the separation part, the received complex bootstrap samples are separated to real and imaginary parts. During this separation, the complex signal is sequentially separated by considering their correlation. The real part, denoted as *Re[t]*, and the imaginary part, denoted as *Im[t]*, represent the real and imaginary components, respectively, of the *t*-th time-domain sample. The length of the separated bootstrap signal is 6144 samples, comprising 3072 real samples and 3072 imaginary samples. The separated data set is inserted to the DNN part. In the DNN, four FC layers operate with the Rectified Linear Unit (ReLU) activation function.

The final FC layer produces a K + 2 dimensional vector, which is subsequently linked to the input of the detection part. The softmax function is applied to calculate the final output in the detection part, representing the value of the time offset estimation. Detailed parameters are listed in Table 3. The estimated time offset value is determined by the maximum value within the softmax output. If the estimated value is *K* + 1, it indicates the absence of the 1st bootstrap symbol in the received signal. Conversely, when detecting for any other value, it implies the detection of a bootstrap signal with *K* samples delayed.

The decision process according to the detected result value *k* can be written as follows:

(4) $$k=\left\{\begin{array}{ll} 0, & \mathrm{detection}\;\mathrm{without}\;\mathrm{time}\;\mathrm{offset}\\ 1\leq k\leq K, & \mathrm{detection}\;\mathrm{with}\;\mathrm{time}\;\mathrm{offset}\;k\\ K+1, & \mathrm{no}\;\mathrm{bootstrap}. \end{array}\right.$$

In this paper, *K* is set to 1000 as defined in Table 3. Therefore, the proposed method can detect the bootstrap symbol with up to 1000 samples of time offset. If the time offset value is greater than 1000 or if the payload portion is received, the value of *k* is determined to be 1001, indicating that there is no first bootstrap symbol. Consequently, the proposed DNN-based bootstrap detector is capable of detecting both the bootstrap symbol and the corresponding time-offset value.

### 3.2. Emergency Wake-Up Signal Demodulator Based on Deep Learning

**Figure 6:**
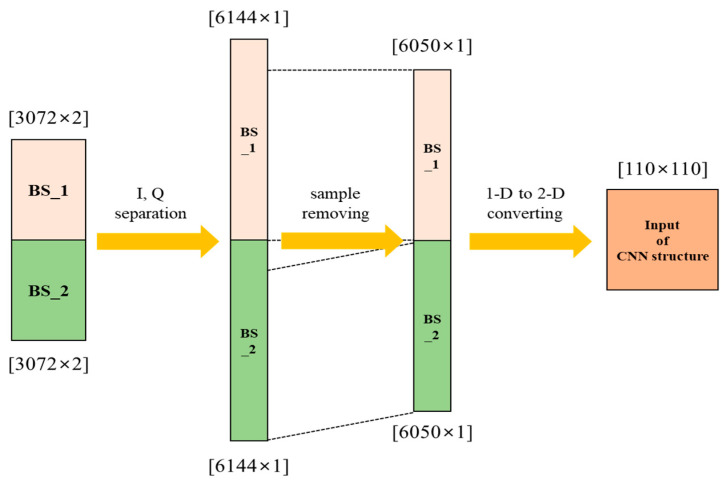
The 2-D representation of the bootstrap symbols for the CNN structure.

**Figure 7:**
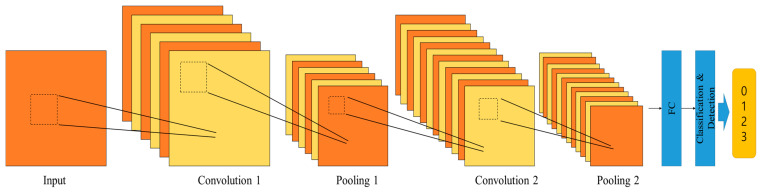
CNN structure for the proposed wake-up signal demodulation.

After bootstrap detection and time-offset estimation, the 2nd and 3rd bootstrap symbols (denoted as BS_1 and BS_2) received without any time-offset are chosen for the demodulation of the emergency wake-up bits. Initially, these selected bootstrap symbols are separated to the real and imaginary parts. In this process, the hardware structure can be simplified by utilizing the same bootstrap symbol detector process. The rearranged symbols, following separation, are organized into a square two-dimensional (2-D) format to conform to the constraints by the proposed CNN structure as described in Figure 6. The matrix (x, y) for re-arranged signal is computed as follows:

(5) $$x,y=\lfloor \sqrt{3072\times 2\times 2}\rfloor =110$$

where, the numerical value 3072 denotes the number of the single bootstrap and the initial ‘2’ corresponds to the separation process of real and imaginary parts. The subsequent ‘2’ represents the representation of the 2nd to 3rd bootstrap symbols. In accordance with (5), a total of 188 samples are required to be discarded during the converting from 1-D to 2-D representation, and the conversion is performed by removing the front part of the signal after I, Q separation. Figure 7 shows the CNN structure for the proposed wake-up signal demodulation.

The proposed CNN architecture is comprised of two distinct layers of convolution and pooling, ultimately linked by an FC layer. The configuration of these layers enhances the network’s ability to extract hierarchical features from input bootstrap symbols. Unlike the bootstrap symbol detector based on DNN, the reason for using the CNN structure is to process a lot of input signal at once and accurately demodulate the emergency wake-up signal. ReLU is used as the activation function in each convolutional layer, with a fixed filter size of 11 × 11. The max pooling operation is performed in the pooling layer for activation. Detailed parameters of the CNN-based wake-up detector is summarized in Table 4.

After passing through the FC layer and undergoing the softmax classifier and one-hot encoding processes, the resulting vector with a fixed length of 4 is output from the detection layer. This vector represents the demodulation result of the emergency wake-up bits. Section 2 details the utilization of two bits for the emergency wake-up function, resulting in four distinct statuses: 0 (00), 1 (01), 2 (10), and 3 (11). The output vector is then matched to these four statuses of the emergency wake-up bits, facilitating efficient signal detection in emergency scenarios. We introduce both DNN and CNN structures considered for bootstrap detection and emergency wake-up signal detection. The parameters utilized in the DNN architecture are detailed in Table 3. Specifically, the DNN structure incorporates four FC layers. ReLU serves as the activation function for the first three FC layers and the final output size is 1002 as mentioned in Section 3. Consequently, the DNN-based bootstrap detector is capable of estimating time-offset values ranging from 0 to 1000 samples, enhancing its utility in signal detection and synchronization tasks. The detailed parameter configuration and architecture design of the CNN-based bootstrap detector is depicted in Table 4. The CNN structure comprises two CNN layers accompanied by two pooling layers. In the filtering process of CNN layers, an 11 × 11 size filter with a stride of 1 and no padding is considered. Similarly, the pooling layers utilize a 2 × 2 size filter with a stride of 1 and no padding. The utilization of a stride of 1 in filtering allows for the seamless processing of multiple data points. At the detection layer, the output consists of four values corresponding to the wake-up signal in four different states, enabling precise signal detection and classification.

## 4. Simulation Results and Discussions

This paper employs the digital terrestrial television broadcasting channel model for experiments with computer simulations, as cited in [13,20]. This channel named RC20 represents a scenario where the signal is exclusively received through the Rician fading with 20 reflected paths where it guarantees a stable direct path from a main transmitter. The channel model is defined as follows: the profile of the RC20 channel is depicted in Table 5, and the received signal in the time domain through the channel is expressed by (6). The proposed deep learning-based method demonstrates the detection of the wake-up signal without compensating for these channels.

(6) $$y[t]=\frac{\rho_{0}x[t]+\sum_{i=1}^{N}\rho_{i}e^{-j\theta_{i}}x[t-\tau_{i}]}{\sqrt{\sum_{i=0}^{N}\rho_{i}^{2}}}\;\;\;\;\;$$

where x[t] is the input signal and N denotes the number of echoes, which is set to 20. Additionally, $\rho_{i},\tau_{i}$ and $\theta_{i}$ represent the attenuation, relative delay and phase shift from scattering of the *i*-th path as listed in Table 5, respectively. The Rician factor *K*, defined as the ratio of the power of the direct path (line-of-sight ray) to that of the reflected paths, is expressed as follows:

(7) $$K=\frac{\rho_{0}^{2}}{\sum_{i=1}^{N}\rho_{i}^{2}}.$$

When the Rician factor *K* is set to 10 dB, the attenuation for this case is as follows:

(8) $$\rho_{0}=\sqrt{10\cdot \sum_{i=1}^{N}\rho_{i}^{2}}.$$

In this simulation, the following ATSC 3.0 system parameters [9] are considered for the 14 bits excluding the emergency alert wake up field used when generating the bootstrap symbols.

- The minimum time interval to the next frame: 100 ms.
- System bandwidth: 6 MHz.
- Sample rate of post-bootstrap: 6.912 MHz.

According to the aforementioned system parameters, the bootstrap signal and ATSC 3.0 payload signal is generated for performance evaluation. The generated signal is received through the fading channel and AWGN as shown in (1). Subsequently, the received signal is fed into the deep learning-based bootstrap detector. The proposed method is entirely conducted on the time domain without utilizing FFT. 

For the training process, the following SNR environments are considered for the proposed deep learning-based emergency alert wake-up signal detection method:

- Bootstrap signal detection: SNR = [−19, −16, −13 dB].
- Wake-up signal detection: SNR = [−22, −19, −16 dB].

**Figure 8:**
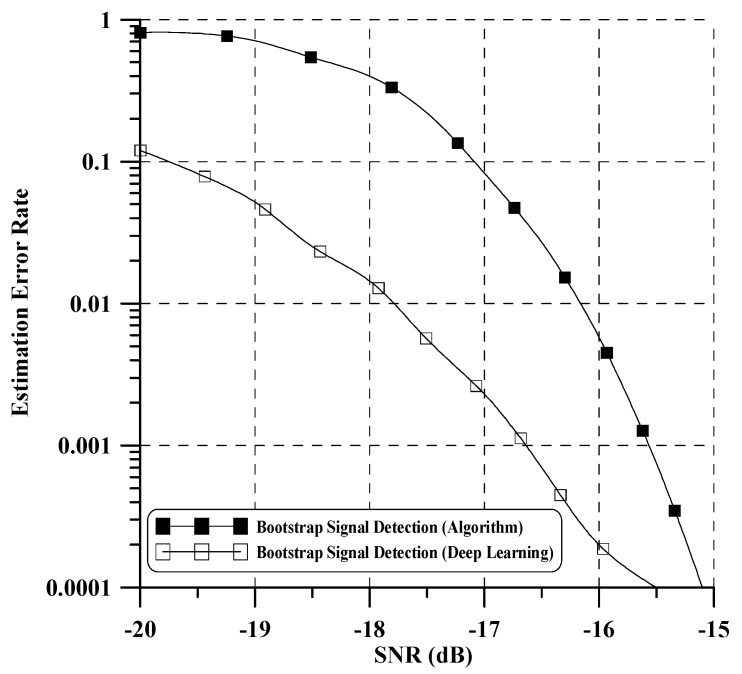
Bootstrap detection error rate performance of proposed method.

**Figure 9:**
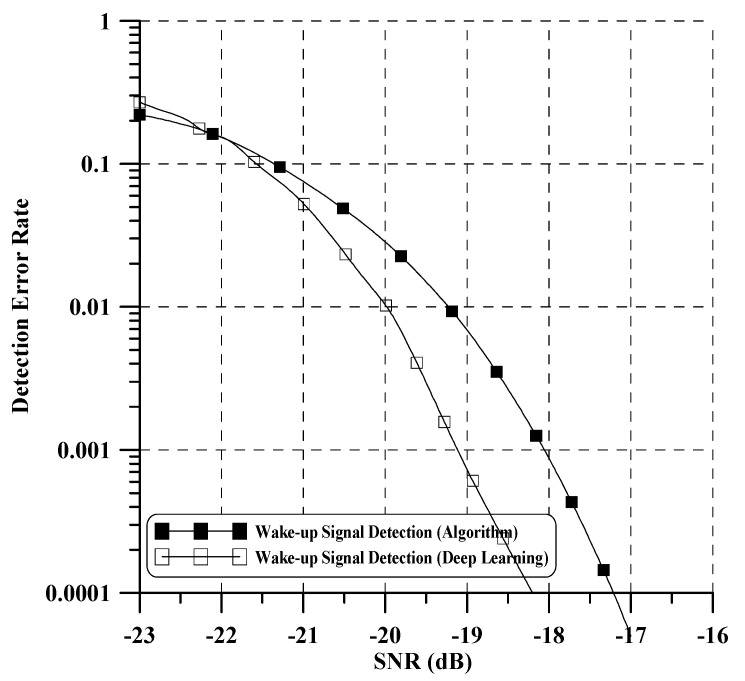
Wake-up signal detection error rate performance of proposed method.

In our test, if any training SNR with near BER = $10^{-2}$ is chosen, the learning process of all considered deep learning architectures can give a rational performance. In addition, training data augmentation is performed by oversampling at four times the symbol rate and shift decimation. This approach provides a more diverse set of examples, helping the model generalize better to unseen instances and enabling it to learn more representative features, thereby leading to improved performance on real-world data. Finally, each DNN and CNN model is trained using 100,000 datasets with a learning rate of 0.005 considered at epoch 30 and a batch size of 100. Figure 8 illustrates the bootstrap detection performance of the proposed method. To benchmark its performance, we compare it with the performance of an existing bootstrap detection technique as referenced in [10]. The results indicate a significant improvement in performance compared to the existing iterative algorithm-based detection technique [10]. Notably, the proposed method demonstrates robust detection of the bootstrap signal across all SNR ranges. Moreover, it is capable of detecting both the bootstrap signal and the time-offset value simultaneously. Figure 9 illustrates the detection error rate performance of the wake-up bits, where the detection error rate signifies the errors encountered when missing all two wake-up bits. For performance evaluation, we compare our proposed method with an existing algorithm-based wake-up bit detection method as outlined in [4]. In low SNR, the performance of our proposed method exhibits a slight decrease compared to the existing method. However, in a high SNR case, our proposed method surpasses the performance of [4]. Notably, our proposed method demonstrates approximately a 1 dB enhancement in performance compared to the algorithm-based method when the detection error rate reaches $10^{-4}$. We confirm that learning through various SNR and oversampled datasets yields a good wake-up signal detection. Additionally, through this performance evaluation, we can further verify that the time offset obtained from the previous bootstrap detection was well compensated. Lastly, the proposed technology applying deep learning has several advantages over existing signal processing technologies:

- It effectively models intricate nonlinear relationships, including channel characteristics, interference, noise, and other factors.
- It learns the interactions between system components, leading to more efficient optimization and improved overall system performance.

**Figure 10:**
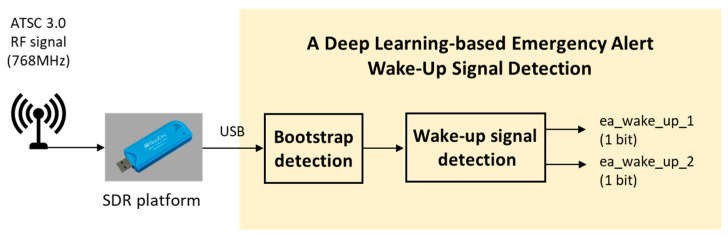
The receiver structure of experiments to verify the proposed method.

Next, this paper conducts additional experiments utilizing the ATSC 3.0-based RF signal and a commercial Software-Defined Radio (SDR) platform to assess the effectiveness of the proposed deep learning-based method in a real environment. The receiver structure of experiments in Figure 10 were designed to validate the performance of our proposed method under realistic conditions. The DekTec DTU-315 device is used as the transmitter for generating the ATSC 3.0 RF signal. The transmitted signals were received using a commercial SDR platform named the Nooelec 820T2. This platform provides functions such as analog-to-digital conversion (ADC), down-conversion, and digital filtering. The experiments parameters are set as follows:

- Center frequency: 768 MHz.
- Channel bandwidth: 6 MHz.
- ADC sampling rate: 1.536 MHz.

**Figure 11:**
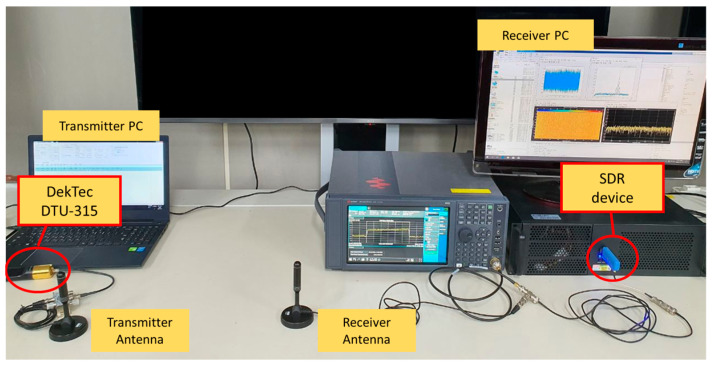
Laboratory test setup.

**Figure 12:**
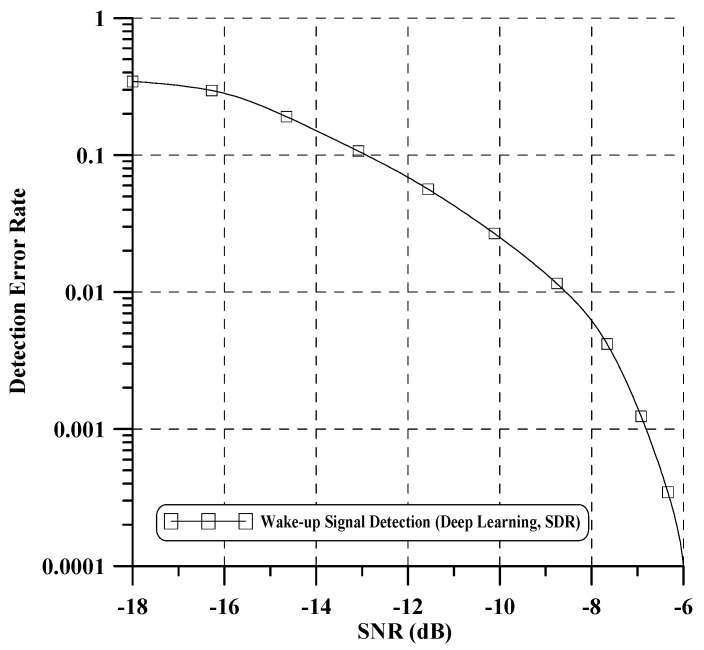
Wake-up signal detection error rate performance in a real environment.

In this setup, the Nooelec 820T2 device supports a maximum sampling rate of 2560 kHz. Therefore, we used a sampling rate of 1.536 MHz, which is one-quarter of the ATSC 3.0 sampling clock rate of 6.144 MHz. The laboratory test environment applying the above parameters is shown in Figure 11 and Figure 12 which shows the wake-up signal detection performance of the proposed deep learning-based method in a real environment. Through this performance validation, we have demonstrated that the proposed deep learning-based technique effectively detects the 2 bits wake-up signal in real environment disaster scenarios. However, compared to the simulated performance in Figure 9, a performance gap of approximately 12 dB is observed. This is attributed to a loss of approximately 6.02 dB due to the ADC sampling rate and additional loss incurred by hardware implementation.

Finally, Table 6 shows a comparison between conventional and proposed methods. The conventional correlation-based method receives input in sample units and performs synchronization using all four bootstrap signals. In contrast, the proposed method conducts bootstrap detection utilizing only the first bootstrap symbol of length 3072 as input for the DNN. The proposed method implements bootstrap demodulation in the time domain without employing channel compensation. By omitting the FFT step, this approach simplifies the procedure. Additionally, it applies CNN instead of the conventional maximum likelihood (ML) decision based on absolute cyclic shift. However, the proposed method has the limitation of acquiring the 2 bits wake-up signal, not the entire bootstrap demodulation signal.

## 5. Conclusions

In this paper, a deep learning-based emergency alert wake-up signal detection method is proposed for the ATSC 3.0 UHD TV system. The wake-up bits, transmitted via the bootstrap signal, serve to notify the public of emergency alert situations. Consequently, the accurate and rapid detection of these wake-up bits holds significant importance for safeguarding lives and property. The proposed method exhibits enhanced performance in detecting the bootstrap symbol and demodulating the wake-up bits. After the training process, the simplicity of the proposed method’s operations enables fast detection of emergency alert situations. Furthermore, the designed deep learning-based detector can identify and demodulate the emergency alert wake-up signal without requiring an ATSC 3.0 demodulator. In conclusion, the proposed method presents a novel approach to wake-up signal detection in ATSC 3.0 emergency alert systems, leveraging deep learning techniques. By employing DNN and CNN, we effectively extract complex patterns and relationships from the received signal. Our approach addresses limitations of traditional methods by operating directly in the time domain, eliminating the need for full demodulation and enabling efficient detection on resource-constrained devices. Furthermore, our proposed method opens up new possibilities for wake-up signal decoding, offering receivers greater flexibility in selecting decoding mechanisms. The proposed method can be implemented in set-top boxes or dedicated disaster receivers. It can also be applied to affordable SDR platforms, allowing for integration with other devices. This flexibility contributes to improved system performance and improves quick recognition of disaster situations.

## Figures and Tables

**Table 1:** Signaling fields for bootstrap symbols.

	Syntax	No. of Bits	Format
Bootstrap Symbol 0 (BS_0)	-	-	-
Bootstrap Symbol 1 (BS_1)	ea wake-up 1	1	uimsbf
	min time to next	5	uimsbf
	system bandwidth	2	uimsbf
Bootstrap Symbol 2 (BS_2)	ea wake-up 2	1	uimsbf
	bsr coefficient	7	uimsbf
Bootstrap Symbol 3 (BS_3)	preamble structure	8	uimsbf

**Table 2:** Fixed OFDM parameters for bootstrap.

Parameters	Value
Sampling rate ($f_{s})$	6.144 Msamples/s
Bandwidth (BW)	4.5 MHz
FFT size ($N_{FFT})$	2048
Subcarrier spacing $\left(f_{∆}\right)$	3 kHz
OFDM symbol duration ($T_{symbol})$	500 μs

**Table 3:** Parameters of the DNN-based bootstrap detector.

Layer	Size	Activation
Input layer	6144	-
FC layer 1	6144	ReLU
FC layer 2	3072	ReLU
FC layer 3	1536	ReLU
FC layer 4	1002	None
Detection	1002 : decision process is as Equation (4)	Softmax

**Table 4:** Parameters of the CNN-based wake-up bit detector.

Layer	Size	Filter Size	Activation
Input layer	110 × 110	-	-
Conv. 1	32@110 × 110	11 × 11	ReLU
Pool. 1	32@55 × 55	2 × 2	Max
Conv. 2	64@55 × 55	11 × 11	ReLU
Pool. 2	64@28 × 28	2 × 2	Max
FC layer	50,176	-	None
Detection	4	-	Softmax

**Table 5:** Channel profile for RC20 channel.

i	$\rho_{i}$	$\tau_{i}\;[\mu s]$	$\theta_{i}\;[rad]$
1	0.95346	0	0
2	0.01618	1.003019	4.855121
3	0.04963	5.442091	3.419109
4	0.11430	0.518650	5.864470
5	0.08522	2.751772	2.215894
6	0.07264	0.602895	3.758058
7	0.01735	1.016585	5.430202
8	0.04220	0.143556	3.952093
9	0.01446	0.153832	1.093586
10	0.05195	3.324866	5.775198
11	0.11265	1.935570	0.154459
12	0.08301	0.429948	5.928282
13	0.09848	3.228872	3.053023
14	0.07380	0.848831	0.628578
15	0.06341	0.073883	2.128544
16	0.04800	0.203952	1.099463
17	0.04203	0.194450	3.462951
18	0.06741	0.924450	3.664773
19	0.03272	1.381320	2.833799
20	0.06208	0.640512	3.334290
21	0.07291	1.368671	0.393889

**Table 6:** Comparison of conventional and proposed methods.

Item		Conventional Method	Proposed Method
Bootstrap Synchronization	input unit	a sample	block (=3072 samples)
	range	4 bootstrap symbols	1st bootstrap symbol
	scheme	correlation	DNN
Channel compensation		O	X
Bootstrap information demodulation	demodulation range	24 bits	2 bits (only wake-up bits)
	scheme	ML decision of the absolute cyclic shift	CNN
	domain	frequency	time

## Data Availability

The data that support the findings of this study are available from the corresponding author upon reasonable request.
